# Translating Tolerogenic Therapies to the Clinic – Where Do We Stand and What are the Barriers?

**DOI:** 10.3389/fimmu.2012.00317

**Published:** 2012-10-12

**Authors:** Stephen P. Cobbold, Xian C. Li

**Affiliations:** ^1^Sir William Dunn School of Pathology, University of OxfordOxford, UK; ^2^Transplant Research Center, Brigham and Women's Hospital, Harvard Medical SchoolBoston, MA, USA

Being able to induce therapeutic tolerance for the treatment of immunological diseases was once considered by many to be an unattainable dream, but recent developments in our understanding of immune regulation and tolerance-compatible immunosuppressive drugs may be turning that dream into reality. Tolerance induction in many rodent models of autoimmune disease and graft rejection is now almost trivial, and the problem has become not how to achieve tolerance experimentally but how to translate this knowledge to real clinical applications in human disease. This volume brings together 17 articles that highlight some of the different approaches being investigated for translating potentially tolerogenic therapies to the clinic and the barriers that still need to be overcome.

The first two articles review the current status of clinical trials that result from large networks of basic laboratories and clinical consortia coordinated by the International Tolerance Network (Page et al., 2012) and the European Framework Program (Issa and Wood, 2012). These networks encompass an enormous range of trials including the minimization of immunosuppressive drugs in organ transplantation, the induction of mixed hematopoietic chimerism without myeloablation, and the use of a variety of antibody and cell therapies that attempt to elicit immune regulation in autoimmune disease, and they vary in scope from small pilot studies to large scale phase III clinical trials. In addition, patients who do achieve long term graft survival or remissions are being analyzed in order to try and determine potential signatures or biomarkers to indicate whether and when, during the course of therapy, immunological tolerance has been established.

The next three articles focus on attempts to exploit immune mechanisms, either by generating regulatory T cells (Treg) *in vitro* for subsequent direct administration to patients, or by utilizing the inherent ability of appropriately differentiated dendritic cells to present antigens for the induction of tolerance and Treg *in vivo*. Sagoo et al. (2012) discuss the issue of the antigen specificity of Treg required for achieving full tolerance to alloantigens, particularly with respect to the issue of direct and indirect antigen presentation. The following two reviews focus on different aspects of tolerogenic antigen presenting cell-based therapies for the induction of immune regulation within the patient. Lutz (2012) focuses on the differentiation status of the dendritic cell required to present antigen for tolerance, Moreau et al. (2012) make a case for the use of autologous, donor antigen pulsed “TolDC.” Together, they highlight the promise of “cell therapy” in transplantation and autoimmunity.

The following six articles consider various novel approaches to tolerance induction. Becker et al. (2012) argue that as we gain a better understanding of the mechanisms by which Treg are induced we should reconsider the clinical use of monoclonal antibodies that target CD4, as these are proving to be particularly effective in a whole range of rodent models of transplantation and autoimmunity. Hamad et al. (2012) suggest that the side effects that so far have limited translation of CD3 or CD20 antibody treatments in Type 1 Diabetes to the clinic (i.e., cytokine release, immunosuppression and EBV proliferation) could be avoided by targeting the FasL molecule, based on the resistance of mice that carry mutations in the Fas pathway of apoptosis to this autoimmune disease. In a different vein, Hirayama et al. (2012) discuss how the naturally acquired tolerance to non-inherited maternal antigens (NIMA) due to reciprocal microchimerism between the mother and fetus during pregnancy may be exploited to limit the risk of graft versus host disease in the choice of donors for bone marrow transplantation. Staying in the arena of hematopoietic transplantation, Carvalho et al. (2012) tackle the question of fungal infections and how tolerogenic process may be required to limit the pathology caused by such infections in transplant recipients and how the interplay of anti-fungal and anti-allo responses may impact on the balance of effector cells and Treg generated. Andreev et al. (2012) similarly discuss this effector/regulatory balance in the lung in the context of asthma when compared to cancer. A paper by Mannie et al. (2012) describes how covalently coupling myelin derived peptide antigens to specific cytokines can target the antigen, presumably via the cytokine receptors, for presentation in a tolerogenic context in rodent models as a potential treatment for inflammatory demyelinating diseases such as multiple sclerosis.

The final six articles discuss in more detail some of the barriers that remain to clinical translation of tolerogenic therapies to the clinic. Pasquet et al. (2011) question the long held assumption derived from classical neonatal tolerance that achieving hematopoietic chimerism is sufficient for full tolerance and provide evidence that Treg activity is also required. Costimulation blockade has long been recognized as a means to induce tolerance and Treg with negative or coinhibitory signaling thought to be important for the maintenance of anergy and Treg activity. McGrath and Najafian (2012) highlight the increasing complexity and redundancy of these multiple pathways and how this provides both challenges and potential opportunities in attempts to target them therapeutically. A particular barrier to the translation of traditional costimulation blockade is the presence of memory T cells, as discussed in the article by Krummey and Ford (2012). Tolerance in rodent models is often induced in mice where naïve T cells predominate, having been maintained in a low pathogen environment while treatments for autoimmune disease are often tested at, or just after, disease induction. Real transplant and autoimmune patients, however, often have high frequencies of memory T cells generated either by heterologous immunity to previous pathogens or due to extended periods of autoreactive inflammatory disease. While memory T cells are often not dependent on CD154/CD40 costimulation, the authors suggest that the addition of LFA-1 or VLA-4 blockade may be a way to overcome this barrier. The final three papers discuss further, often ignored, potential difficulties for realizing tolerogenic therapies: the activation of the innate immune system, in particular NK cells (Benichou et al., 2012), the impact of lymphoid trafficking of regulatory and effector T cells to and from tissues (Burrell et al., 2011), and the interaction of the immune system with the tissue microenvironment, especially the vasculature (Bruneau et al., 2012).

In summary, we have made considerable progress toward translating potentially tolerogenic therapies from rodent models into the clinic, to the point where we now have a much clearer understanding of the barriers that remain and how we may yet overcome them.
